# Effect of Concurrent Chemoradiation With Celecoxib vs Concurrent Chemoradiation Alone on Survival Among Patients With Non–Small Cell Lung Cancer With and Without Cyclooxygenase 2 Genetic Variants: A Phase 2 Randomized Clinical Trial

**DOI:** 10.1001/jamanetworkopen.2019.18070

**Published:** 2019-12-18

**Authors:** Nan Bi, Jun Liang, Zongmei Zhou, Dongfu Chen, Zhixue Fu, Xu Yang, QinFu Feng, Zhouguang Hui, Zefen Xiao, Jima Lv, Xiaozhen Wang, Tao Zhang, Xin Wang, Lei Deng, Wenqing Wang, Jingbo Wang, Lipin Liu, Chen Hu, Luhua Wang

**Affiliations:** 1 Department of Radiation Oncology, National Cancer Center and Cancer Hospital, Peking Union Medical College, Chinese Academy of Medical Sciences, Beijing, China; 2 Division of Biostatistics and Bioinformatics, Sidney Kimmel Comprehensive Cancer Center, Johns Hopkins University School of Medicine, Baltimore, Maryland; 3 Department of Radiation Oncology, Shenzhen Hospital, Peking Union Medical College, Chinese Academy of Medical Sciences, Beijing, China

## Abstract

**Importance:**

Treatment of locally advanced non–small cell lung cancer (NSCLC) remains challenging. The rationale of combining a cyclooxygenase 2 (COX-2) inhibitor with concurrent chemoradiation (CCRT) was based on results of preclinical research and prospective clinical studies; however, no randomized clinical trial has provided evidence of a direct comparison with CCRT alone.

**Objective:**

To determine the effect of combined selective COX-2 inhibition with standard CCRT on survival among patients with unresectable stage III NSCLC.

**Design, Setting, and Participants:**

A single-center, open-label, randomized phase 2 clinical trial was performed among 96 patients who had histologically and cytologically confirmed unresectable stage III NSCLC. Participants were enrolled from November 2011 to August 2015. Data were analyzed from February to October 2018.

**Intervention:**

Patients were randomized to receive thoracic radiation, 60 Gy, for 6 weeks concurrent with etoposide and cisplatin or the same regimen of CCRT combined with 200 mg of celecoxib, taken twice daily.

**Main Outcomes and Measures:**

The primary end point was overall survival. The secondary end points were the proportion of patients with treatment-related toxic effects, progression-free survival, and overall survival in subgroups with and without the COX-2 genotype.

**Results:**

A total of 100 patients were randomized. Following the exclusion of 4 outliers, 96 participants (96.0%) were analyzed (51 randomized to CCRT alone and 45 randomized to CCRT with celecoxib; mean [SD] age, 60.0 [8.3] years; 73.0 [76.0%] male). The median overall survival time was 32.8 (95% CI, 17.0-48.5) months in the group that received CCRT with celecoxib and 35.5 (95% CI, 25.8-45.2) months in the group that received CCRT alone (*P* = .88). Celecoxib with CCRT was well tolerated; the incidence of symptomatic radiation pneumonitis was 6.6% (95% CI, 1.4%-18.0%) in the group that received CCRT with celecoxib and 11.8% (95% CI, 4.4%-23.9%) in the group that received CCRT alone (*P* = .49). Among patients with the high-risk genotype, celecoxib plus CCRT was not associated with higher progression-free survival (hazard ratio, 0.36; 95% CI, 0.13-1.04; *P* = .05) or overall survival (hazard ratio, 0.50; 95% CI, 0.15-1.72; *P* = .26) compared with CCRT alone.

**Conclusions and Relevance:**

In unresectable stage III NSCLC, adding celecoxib to concurrent chemoradiation did not improve survival. A smaller, not statistically significant proportion of patients in the CCRT with celecoxib group compared with the CCRT alone group developed symptomatic radiation pneumonitis. Among patients with the high-risk genotype, adding celecoxib to CCRT did not improve overall or progression-free survival.

**Trial Registration:**

ClinicalTrials.gov identifier: NCT01503385

## Key Points

**Question:**

Could selective cyclooxygenase 2 inhibition combined with standard concurrent chemoradiation therapy improve survival among patients with unresectable stage III non–small cell lung cancer?

**Findings:**

In this phase 2 clinical trial of 96 patients, adding celecoxib to concurrent chemoradiation did not improve survival.

**Meaning:**

Adding celecoxib to concurrent chemoradiation did not improve survival.

## Teaser

This phase 2 randomized clinical trial evaluates the effect of combined selective cyclooxygenase 2 (COX-2) inhibition with standard concurrent chemoradiation therapy vs concurrent chemoradiation alone on survival among patients with unresectable stage III non–small cell lung cancer (NSCLC).

## Introduction

Approximately 30% of patients with non–small cell lung cancer (NSCLC) have locally advanced disease.^1^ Although concurrent chemotherapy and radiation is considered standard care,^2^ treatment of locally advanced NSCLC remains challenging, with a survival rate of less than 20% at 5 years.^3^ Novel agents and strategies are warranted to maximize treatment effects and reduce toxic effects to healthy tissue.

The overexpression of cyclooxygenase 2 (COX-2) has been reported in NSCLC.^4,5^ Increased COX-2 expression is associated with more aggressive tumor behavior and poorer prognosis in patients with NSCLC.^6,7,8,9^ The rationale for combining a selective COX-2 inhibitor with CCRT was based on the results of preclinical research and nonrandomized clinical studies. Selective COX-2 inhibitors could enhance tumor radiosensitivity in cell and animal models,^10,11,12^ which was only observed among patients with tumors expressing COX-2. Several prospective, single-arm clinical trials showed that adding a COX-2 inhibitor to thoracic radiotherapy with or without chemotherapy for inoperable NSCLC could improve outcomes without increasing toxic effects to healthy tissue,^13,14,15^ which highlighted the potential benefit of this novel strategy. However, to our knowledge, no randomized clinical trial has provided any results of direct comparisons.

Radiation-induced pneumonitis is the most common dose-limiting complication in patients with lung cancer who are treated with thoracic radiotherapy. This adverse effect remains a concern when chemotherapy is combined with thoracic radiotherapy. Since COX-2 plays an important role in prostaglandin production of inflammatory processes occurring in irradiated tissues, COX-2 inhibitors, as a type of nonsteroidal anti-inflammatory drug, might sensitize tumors to concurrent radiation and mitigate radiation-induced lung injury at the same time. It has been demonstrated that celecoxib, a selective COX-2 inhibitor, could significantly reduce lung toxic effects in mice and could be clinically useful.^16^

It is critical to identify patients who may benefit from COX-2–targeted therapy. We previously reported that single-nucleotide polymorphism (SNP) −1195G>A (rs689466) in the COX-2 promoter region was associated with survival advantage in inoperable, locally advanced NSCLC treated with chemoradiation or radiation alone.^7^ Tumors carrying unfavorable −1195dupA genotypes were more resistant to radiation than those with the −1195GA or −1195GG genotypes, which might require more intensive treatment. Functional studies demonstrated that patients with the −1195dupA genotype had significantly increased COX-2 expression compared with their counterparts with −1195GA or −1195GG genotypes and thus might benefit more from COX-2–targeted therapy.

On the basis of these results, we performed a randomized phase 2 clinical trial to determine the value of combined selective COX-2 inhibition with standard concurrent chemoradiation therapy (CCRT) among patients with unresectable stage III NSCLC, with a focus on survival, treatment-related lung toxic effects, and the association of the COX-2 and −1195G>A polymorphism with overall survival (OS).

## Methods

This was a prospective, randomized, open-label, single-center phase 2 trial. The control group received standard care. Eligible patients were assigned to receive CCRT either with or without celecoxib by simple randomization. The random table method was used to generate a random number sequence by 1 of us (J. Liang). The randomization was concealed using the sealed, opaque envelope method until the patient entered the study. Patients were enrolled from November 2011 to August 2015. The trial protocol, which appears in Supplement 1, was approved by the ethical review board of the National Cancer Center and Cancer Hospital. All patients provided written informed consent prior to enrollment. The current study followed the Consolidated Standards of Reporting Trials (CONSORT) reporting guideline as well as the Strengthening the Reporting of Observational Studies in Epidemiology (STROBE) reporting guideline.

### Patient Eligibility

Patients were required to have histologically and cytologically confirmed stage III NSCLC. Eligible patients were also required to meet the following criteria: (1) aged between 18 and 70 years; (2) had an Eastern Cooperative Oncology Group performance status of 1 or less; (3) experienced no more than 10% weight loss in the 3 months before inclusion; (4) had inoperable stage IIIA or IIIB NSCLC, per the American Joint Committee on Cancer; and (5) had normal organ function. Ineligibility criteria included active uncontrolled infection; clinically significant cardiovascular disease; history of other malignant neoplasms; forced expiratory volume in 1 second of less than 40% of baseline; and previous treatment with radiotherapy, chemotherapy, or immunotherapy.

### Treatment

The CCRT regimen was identical in both arms. In the concurrent schedule, chemotherapy consisted of 50 mg/m^2^ of etoposide on days 1 to 5 and 50 mg/m^2^ of cisplatin on days 1 and 8, every 4 weeks (EP regimen) for 2 cycles.^17,18^ All patients underwent simplified intensity-modulated radiotherapy.^18^ The gross tumor volume included the primary disease as well as any involved regional lymph nodes, which were defined as those with a short-axis diameter of at least 1 cm on computed tomography (CT) scan or with high fluorodeoxyglucose uptake on positron emission tomography–CT scan. The clinical tumor volume included primary tumor plus a margin of 0.6 to 0.8 cm, with ipsilateral hilum and mediastinal nodal stations involved. A dose of 60 Gy (ie, 2 Gy per fraction) started on the first day of chemotherapy. The mean dose to the lungs should optimally be 17 Gy or less; the lung volumes, minus gross tumor volume, receiving more than 20 Gy and 30 Gy were limited to less than 30% and less than 20%, respectively.

Patients were randomly assigned to CCRT alone or CCRT with celecoxib. Celecoxib, at 200 mg twice daily, was started 1 week before the initiation of radiotherapy and was continued without interruption until the end of radiotherapy.^13^

Because consolidation chemotherapy after CCRT is not standard care and no strong data support the use of it, delivering platinum-based doublet consolidation chemotherapy by medical oncologists, per local protocol, was permitted. Either platinum-based doublet chemotherapy regimen or single-agent chemotherapy regimen was acceptable.

### Evaluation and Follow-up

Pretreatment evaluation included chest and abdominal CT scans, brain magnetic resonance imaging scans and CT scans, bronchoscopies, and radionuclide bone scans. It was recommended that participants undergo positron emission tomography–CTs, but they were not mandatory.

The follow-up evaluations consisted of patient history, a physical examination, and a thoracic CT at intervals of 3 months for 2 years and then at intervals of 6 months (or earlier if clinically indicated) for 3 years. Other imaging examinations were obtained when recurrence was suspected.

The treatment response was evaluated using the Response Evaluation Criteria in Solid Tumors version 1.0. Toxic effects were graded according to the National Cancer Institute Common Toxicity Criteria version 4.0.

### SNP Genotyping

Genomic DNA was extracted from a 1-mL blood sample that was collected at baseline. Each specimen was stored at −80 °C. The COX-2 −1195G>A polymorphisms were genotyped as previously described^7^ by the MassARRAY compact system (Agena Bioscience) in a blinded manner. In brief, DNA was extracted by a QIAamp DNA Mini Kit (Qiagen), and multiplex polymerase chain reaction was conducted for DNA amplification followed by dephosphorylation with alkaline phosphatase and primer extension reactions. The products were spotted onto the MassARRAY SpectroCHIP (Agena Bioscience) and detected using matrix-assisted laser desorption ionization–time of flight. The results were analyzed using Typer version 4.0 software (Agena Bioscience). The high-risk genotype was the −1195dupA homozygote, and the low-risk group included −1195GA and −1195GG genotypes.

### Statistical Analysis

The primary end point of this trial was OS. We reported a 2-year OS rate of 48% for CCRT with etoposide and cisplatin.^18^ Liao et al^13^ reported a 2-year OS rate of 67% with CCRT with celecoxib. The power analysis and sample size estimation were completed using the log-rank test. Assuming 10% of patients would be lost to follow-up with 50 patients per arm (ie, a total of 100), observing at least 59 deaths provides 85% power to detect 19% superiority (67% vs 48%) in OS at 2 years from randomization (ie, equivalent to a hazard ratio of 0.55) with a 1-sided type I error rate of 0.1.

The secondary end points were the proportion of patients with treatment-related toxic effects, progression-free survival (PFS), and OS in COX-2 genotype subgroups. We calculated OS from the day of randomization to death or last follow-up (including study cutoff) if still alive. We calculated PFS from randomization to death or progression, whichever occurred first. The rates of OS and PFS were estimated using the Kaplan-Meier method and compared with the log-rank test. To minimize any potential imbalance in patient characteristics because of the small sample size, their distributions were compared with *t* tests, nonparametric Mann-Whitney *U* tests, or χ^2^ tests. Unadjusted and adjusted hazard ratios (HRs) were estimated using Cox proportional hazard models. Toxic effect rates and response rates were compared with the Fisher exact test. Estimated treatment effects for CCRT with celecoxib compared with CCRT alone were expressed as HRs with 95% CIs, derived from an unstratified Cox model. Survival analyses were performed in biomarker subgroups for PFS and OS. We also investigated the effect of epidermal growth factor receptor (*EGFR*) gene mutation because of longer survival for these patients receiving EGFR tyrosine kinase inhibitor treatment. Patients with the *EGFR* gene mutation were excluded in post hoc analysis. A 2-sided *P* < .05 was considered statistically significant, except for OS per study design. All analyses were conducted with SPSS statistical software version 17.0 (IBM Corp) and R version 3.4.2 (R Project for Statistical Computing).

## Results

### Patient Characteristics

**Figure 1.**
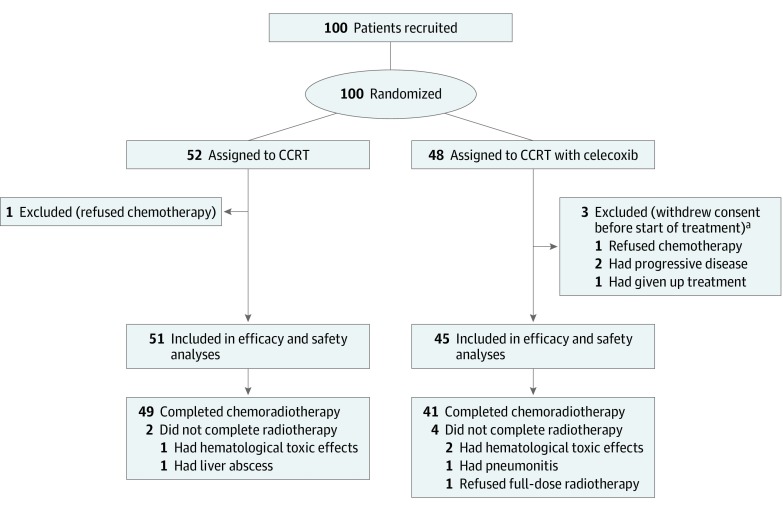
Flow Diagram for the Trial CCRT indicates concurrent chemoradiation therapy. ^a^Patients could have more than 1 reason for exclusion.

The baseline characteristics were well balanced between the 2 arms (Table 1). As shown in Figure 1, 100 patients were randomized. Following the exclusion of 4 outliers, 96 participants (51 in the CCRT group and 45 in the CCRT with celecoxib group) were included in efficacy analyses (mean [SD] age, 60.0 [8.3] years; 73 men [76.0%]). A total of 90 patients (49 in the CCRT group and 41 in the CCRT with celecoxib group) received the definitive treatment with a radiation dose of at least 50 Gy. The median (range) follow-up period was 50.0 (32.2-73.6) months. No patients were lost to follow-up.

**Table 1.** Demographic and Baseline Clinical Characteristics of Patients

Characteristic	No. (%)		*P* Value
	CCRT Group (n = 51)	CCRT With Celecoxib Group (n = 45)	
Age, y			
<65	38 (74.5)	39 (86.7)	.20
≥65	13 (25.5)	6 (13.3)	
Sex			
Men	39 (76.5)	36 (80.0)	>.99
Women	12 (23.5)	9 (20.0)	
ECOG Performance Status			
0	24 (47.1)	25 (55.6)	.23
1	27 (52.9)	20 (44.4)	
Pathology			
Squamous	36 (70.6)	31 (68.9)	.91
Adenocarcinoma	12 (23.5)	12 (26.7)	
Other	3 (5.9)	2 (4.4)	
AJCC stage			
IIIA	20 (39.2)	15 (33.3)	.53
IIIB	31 (60.8)	30 (66.7)	
Smoking history			
Yes	39 (76.5)	34 (75.6)	>.99
No	12 (23.5)	11 (24.4)	

Abbreviations: AJCC, American Joint Committee on Cancer; CCRT, concurrent chemoradiation; ECOG, Eastern Cooperative Oncology Group.

### Treatment Delivery

Celecoxib was given in compliance with guidelines to all the patients in the CCRT with celecoxib arm. As shown in Figure 1, thoracic radiation was given as planned or with minor deviations in 49 patients (96.1%) in the CCRT group and 41 patients (91.1%) in the CCRT with celecoxib group. Patients receiving 2 cycles of concurrent chemotherapy were numerically more allocated to the CCRT with celecoxib group (39 of 45 patients [86.7%]) than the CCRT group (37 of 51 patients [72.5%]), although without statistical significance (*P* = .09). The reasons for not completing concurrent chemotherapy included toxic effects (2 patients in the CCRT group and 3 in the CCRT with celecoxib group) or patient refusal (1 in the CCRT group and 2 in the CCRT with celecoxib group). The proportion of patients receiving consolidation chemotherapy were similar between the 2 groups (49.0% [25 of 52] in the CCRT group vs 51.1% [23 of 48] in the CCRT with celecoxib group; *P* = .84).

### Survival and Response

A total of 61 patients (63.5%) died (33 [64.7%] in the CCRT group and 28 [62.2%] in the CCRT with celecoxib group). As shown in eFigure 1 in Supplement 2, OS was not significantly different between patients who received celecoxib and those who did not (median OS time, 32.8 [95% CI, 17.0-48.5] months vs 35.5 [95% CI, 25.8-45.2] months; *P* = .88). The 2-, 3-, and 5-year OS rates were 66.7%, 48.5%, and 30.3%, respectively, among patients in the CCRT with celecoxib group and 68.9%, 48.1%, and 31.6%, respectively, among patients in the CCRT group. A total of 82 patients (85.4%) progressed (45 [88.2%] in the CCRT group and 37 [82.2%] in the CCRT with celecoxib group) at the last follow-up. There was no difference in median PFS (17.0 [95% CI, 9.9-24.1] months vs 16.0 [95% CI, 10.9-21.0] months; *P* = .38). The 2-, 3-, and 5-year PFS rates were 38.0%, 21.9%, and 16.1%, respectively, among patients in the CCRT with celecoxib group and 25.4%, 17.7%, and 6.8%, respectively, among patients in the CCRT group. The overall complete response rates were 82.2% among patients in the CCRT with celecoxib group and 80.4% among patients in the CCRT group, which showed no significant difference (*P* = .16).

### Toxic Effects

Grade 2 to 5 toxic effects of treatments are listed in Table 2. Both treatment regimens were well tolerated. No grade 5 toxic effects were reported. Hematologic and nonhematologic toxic effects were similar to those demonstrated in prior studies of concurrent chemoradiation. No cardiovascular events, allergic events, or gastrointestinal ulcers occurred during treatment in the CCRT with celecoxib arm. A decrease in the incidence of symptomatic radiation pneumonitis was observed among patients in the CCRT with celecoxib group (6.6%; 95% CI, 1.4%-18.0%) compared with patients in the CCRT group (11.8%; 95% CI, 4.4%-23.9%). However, there was no significant difference because of the low incidence and limited sample size (*P* = .49).

**Table 2.** Treatment-Related Toxic Effects According to Treatment Regimen

Toxic Effect	No. (%)													
	CCRT Group (n = 51)							CCRT With Celecoxib (n = 45)						
	Grade 0	Grade 1	Grade 2	Grade 3	Grade 4	Grade 5	≥Grade 3	Grade 0	Grade 1	Grade 2	Grade 3	Grade 4	Grade 5	≥Grade 3
Esophagitis	25 (49.0)	8 (15.7)	13 (25.5)	5 (9.8)	0	0	5 (9.8)	17 (37.8)	39 (20.0)	17 (37.8)	2 (4.4)	0	0	2 (4.4)
Leukopenia	11 (21.6)	13 (25.5)	6 (11.8)	17 (33.3)	4 (7.8)	0	21 (41.1)	5 (11.1)	11 (24.4)	14 (31.3)	13 (28.9)	2 (4.4)	0	15 (33.3)
Anemia	34 (66.7)	10 (19.6)	6 (11.8)	1 (2.0)	0	0	1 (2)	33 (73.3)	8 (17.8)	2 (4.4)	1 (2.2)	1 (2.2)	0	2 (4.4)
Thrombocytopenia	38 (74.5)	5 (9.8)	4 (7.8)	3 (5.9)	1 (2.0)	0	4 (7.9)	35 (77.8)	7 (15.6)	1 (2.2)	2 (4.4)	0	0	2 (4.4)
Dermatological toxic effects	27 (52.9)	18 (35.3)	6 (11.8)	0 (1.1)	0	0	0	24 (54.5)	17 (38.6)	3 (6.8)	0	0	0	0
Radiation pneumonitis	43 (84.3)	2 (3.9)	3 (5.9)	3 (5.9)	0	0	6 (11.8)^a^	40 (88.9)	2 (4.4)	1 (2.2)	2 (4.4)	0	0	3 (6.6)^a^
Gastrointestinal toxic effects	28 (56.0)	18 (36.0)	3 (6.0)	1 (2.0)	0	0	1 (2.0)	25 (55.6)	10 (22.2)	9 (20.0)	1 (2.2)	0	0	1 (2.2)
Allergic reaction	50 (98.0)	1 (2.0)	0	0	0	0	0	44 (97.2)	1 (2.20)	0	0	0	0	0
Cardiac toxic effects	46 (90.2)	3 (5.9)	2 (3.9)	0	0	0	0	45 (100)	0	0	0	0	0	0
Hemorrhage	50 (98.0)	1 (2.0)	0	0	0	0	0	43 (95.6)	2 (4.4)	0	0	0	0	0

Abbreviation: CCRT, concurrent chemoradiation.

^a^ Includes grade 2 toxic effects.

### COX-2 −1195dupA Genotype and Survival

A total of 63 patients (65.6%) receiving definitive treatment displayed genotyping successfully. The baseline characteristics of groups with and without the biomarker were well balanced, except for a slightly higher proportion of men (eTable in Supplement 2). In patients who had tumors genotyped, there were no significant differences noted for OS (HR, 1.53; 95% CI, 0.85-2.74; *P* = .15) or PFS (HR, 1.30; 95% CI, 0.80-2.11; *P* = .29) (eFigure 2 in Supplement 2).

**Figure 2.**
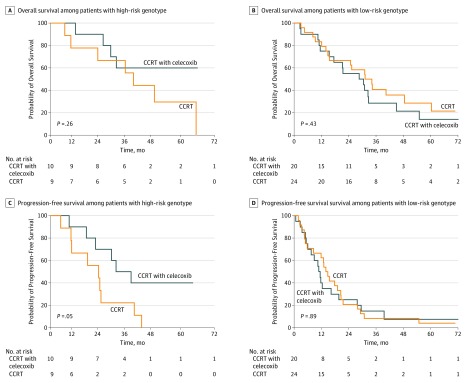
Kaplan-Meier Curves for Overall and Progression-Free Survival Among Patients With High-Risk and Low-Risk Genotypes CCRT indicates concurrent chemoradiation therapy.

A total of 19 patients (30.2%) had high-risk genotypes (9 [17.6%] in the CCRT group and 10 [22.2%] in the CCRT with celecoxib group), and 44 patients (69.8%) had low-risk genotypes (24 [47.1%] in the CCRT group and 20 [44.4%] in the CCRT with celecoxib group). Genotype was not associated with either OS or PFS in the CCRT arm, but it was associated with PFS in the CCRT with celecoxib arm (HR, 0.58; 95% CI, 0.36-0.93; *P* = .02). There was no statistically significant difference between patients with the high-risk genotype receiving CCRT with celecoxib and patients receiving CCRT alone in PFS (HR, 0.36; 95% CI 0.13-1.04; *P* = .05) or OS (HR, 0.50; 95% CI, 0.15-1.72; *P* = .26). There was no statistically significant difference between patients with the low-risk genotype who received CCRT with celecoxib and those who received CCRT alone (PFS: HR, 1.05; 95% CI, 0.56-1.95; *P* = .89; OS: HR, 1.32; 95% CI, 0.67-2.62; *P* = .43) (Figure 2). The interaction of treatment effect (CCRT with celecoxib vs CCRT alone) and COX-2 genotypes (high-risk vs low-risk) from multivariable Cox regression analysis was not significant (HR, 0.87; 95% CI, 0.72-1.06; *P* = .10) because of the small number of patients.

After the exclusion of 3 patients with *EGFR* gene mutations, there were 18 patients (30.0%) with high-risk genotypes (8 [15.6%] in the CCRT group and 10 [22.2%] in the CCRT with celecoxib group), and 42 patients (70.0%) with low-risk genotypes (24 [47.1%] in the CCRT group and 18 [40.0%] in the CCRT with celecoxib). As shown in eFigure 3 in Supplement 2, genotype was not associated with OS or PFS in either group (high-risk genotype, PFS: HR, 0.37; 95% CI, 0.13-1.09; *P* = .06; OS: HR, 0.48; 95% CI, 0.13-1.70; *P* = .24; low-risk genotype, PFS: HR, 0.97; 95% CI 0.51-1.85; *P* = .94; OS: HR, 1.29; 95% CI, 0.64-2.64; *P* = .48).

## Discussion

To our knowledge, this study was the first randomized clinical trial designed to directly elucidate whether inhibiting COX-2 activity could improve outcomes of CCRT in locally advanced NSCLC. Table 3 lists prospective trials that investigated the combination of celecoxib with radiation therapy.^13,14,15^ The results showed that adding celecoxib to concurrent radiation or chemoradiation for inoperable NSCLC might have moderate efficacy without increasing toxic effects in healthy tissue. Similarly, our randomized phase 2 trial failed to confirm the value of the combination of celecoxib at 400 mg/d with CCRT in all patients.

**Table 3.** Randomized Clinical Trials of Radiation Therapy With Celecoxib

Source	Phase	Patients, No.	Stage	RT Dose, Gy	Concurrent Chemotherapy	Celecoxib Dose	Median OS, mo (2-y OS rate, %)	Median PFS, mo (2-y PFS rate, %)	Lung Toxic Effects, Grade (%)
Liao et al,^13^ 2005	1	47	I-IV	45/15F, 66/33F, or 63/35F	No	200-800 mg/d 5-7 d before RT and continued throughout RT	24 (54.6)	NA	2 (10.6); 3 (4.3)
Komaki et al,^14^ 2011	1	20	II/III	63/35F	Irinotecan and cisplatin	10 Patients received 200 mg/d and 10 patients received 400 mg/d	37.5 (65)	NA	Pulmonary fibrosis improved in 400-mg group vs in 200-mg group (*P* = .04)
Gore et al,^15^ 2011	1/2	18	IIB, IIIA/B	45/15F or 60-66/30-33F	No	7 Patients received 400 mg/d and 11 patients received 800 mg/d, 5 d before RT and continued for 2 y or until progression	10 (22.2)	12 (33.3)	>3 (0)
Present study	2	96	IIIA/B	60; 2/F	Etoposide and cisplatin	400 mg/d, 7 d before RT and continued throughout RT	CCRT group, 35.5 (67); CCRT with celecoxib group, 32.8 (69)	CCRT group, 18.2 (25); CCRT with celecoxib, 20 (38)	CCRT group, 2 (11.8); CCRT with celecoxib group, 2 (6.6)

Abbreviations: CCRT, concurrent chemoradiation; F, fraction; NA, not applicable; OS, overall survival; PFS, progression free survival; RT, radiation therapy.

A decrease of symptomatic radiation-induced pneumonitis incidence (6.6% vs 11.8%) was observed among patients in the CCRT with celecoxib arm compared with patients in the CCRT alone arm. However, the difference was not statistically significant because of the small sample size. Hunter et al^16^ previously reported that celecoxib significantly reduced lung toxic effects in mice, suggesting the feasibility of using COX-2 inhibitors to treat radiation-induced lung toxic effects as a complement to concurrent radiation therapy of lung cancers. The median lethal dose for celecoxib-treated mice was significantly higher than for untreated mice (12.9 [95% CI, 12.55-13.25] Gy vs 12.4 [95% CI 12.2-12.65] Gy; *P* = .03).^16^ Komaki et al^14^ found that pulmonary fibrosis was less common in the higher-dose (ie, 400 mg) group than in the lower-dose group. As shown in Table 3, the incidence of symptomatic radiation-induced pneumonitis was between 6.6% and 14.9% after combined celecoxib and radiotherapy with or without chemotherapy. Comparisons with historical data from an international individual-patient data meta-analysis of 836 patients^19^ indicated that this number was dramatically lower than historical controls (29.8%). Although preliminary, our results, together with data from other prospective studies, suggested that there could be a reduced risk of radiation-induced pneumonitis after combined celecoxib and chemoradiation and highlighted the potential role of celecoxib in reducing radiation-induced lung toxic effects.

In the era of molecular targeted therapy, the optimal selection of patients who may benefit from a combined strategy of COX-2 suppression and CCRT appears to be critical. The COX-2 expression level in the tumor is a candidate. However, emerging evidence indicated that it seems inappropriate to use this method for patient selection,^20,21^ which is possibly because of tumor heterogeneity as well as potential subjectivity of immunohistochemistry evaluation. It is also limited by tumor tissue availability. Another promising biomarker is the urinary metabolite of prostaglandin E_2_ level, although the results of different studies remain controversial. Edelman et al^21^ found that baseline urinary prostaglandin E_2_ level was a negative prognostic and possibly a predictive marker in advanced NSCLC, while both Altorki et al^22^ and Edelman et al^23^ failed to validate it.

In this study, a predefined analysis indicated that the COX-2 −1195dupA genotype might be associated with benefit from the combination of celecoxib and CCRT. This genotype was seen in 19 of 63 assessable blood samples (30.2%). We previously identified that the genetic polymorphisms in the COX-2 gene were associated with outcomes in patients with locally advanced NSCLC treated with chemoradiotherapy or radiotherapy alone.^7^ This concept is now confirmed for the first time in a prospective trial. This may explain the negative results of the whole group because the positive effect among patients with the high-risk genotype may have been obscured by the negative effects on those with the low-risk genotype. The functional study^24^ showed that the −1195G>A change created a *C-MYB* binding site in the COX-2 promoter region and, thus, displayed a higher promoter activity. Compared with their counterparts with −1195GG and −1195GA, the patients with −1195dupA showed significantly increased COX-2 expression in vitro and in vivo, showing that functional −1195G>A SNP, which might influence the expression of COX-2, led to decreased survival in NSCLC independently. Vogel et al^25^ revealed a gene-drug interaction between the COX-2 −1195G>A SNP and COX-2 inhibitor use, enhancing lung cancer risk. The COX-2 −1195A allele was also associated with a poor response to vinorelbine-based chemotherapy in patients with NSCLC.^26^ Therefore, it was the rationale that a more intensive treatment, such as a combination of chemoradiation therapy and COX-2 inhibition, could improve the survival in patients with the unfavorable COX-2 −1195dupA genotype.

### Limitations

Limitations of this study include its moderate sample size and retrospective design of biomarker analyses. Therefore, no definitive conclusions could be drawn at present. Further prospective randomized investigations of lung toxic effects and biomarkers within larger sample sizes are warranted. Additionally, because genetic markers are specific to race and ethnicity, our results should be validated among different populations in the future.

## Conclusions

This phase 2 randomized study failed to demonstrate the value of adding a COX-2 inhibitor to CCRT for unresectable, inoperable, locally advanced NSCLC. The treatment was well tolerated and reduced risk of symptomatic radiation pneumonitis by 44%, although the difference was not statistically significant. Our results might be important for designing further clinical trials of COX-2 inhibitors.

## Notes

Supplement 1. **Trial Protocol**

Supplement 2. **eTable. **Patient Characteristics for COX-2 Genotyping **eFigure 1.** Kaplan-Meier Curves by Treatment Arm **eFigure 2.** Kaplan-Meier Curves by Gene Testing **eFigure 3.** Kaplan-Meier Curves in Patients Without *EGFR* Mutations

Supplement 3. **Data Sharing Statement**
