# Supplementary material for: Effect of Concurrent Chemoradiation With Celecoxib vs Concurrent Chemoradiation Alone on Survival Among Patients With Non–Small Cell Lung Cancer With and Without Cyclooxygenase 2 Genetic Variants: A Phase 2 Randomized Clinical Trial
Source: JAMA Netw Open. 2019 Dec 18;2(12):e1918070. doi: 10.1001/jamanetworkopen.2019.18070 (PMC6991217; doi:10.1001/jamanetworkopen.2019.18070)
Supplement: Supplement 1. — Trial Protocol [file jamanetwopen-2-e1918070-s001.pdf]

Cisplatin/Etoposide and Concurrent Radiotherapy With or Without Celecoxib in Patients With Unresectable Locally Advanced Non-small Cell Lung Cancer (NSCLC)

Department of Radiation Oncology, National Cancer Center, Cancer Hospital and Institute, Peking Union Medical College (PUMC) and Chinese Academy of Medical Sciences (CAMS)

PI: Luhua Wang, Email: wlhwq@yahoo.com; Tel: +861087788799

Clinical research site: Department of Radiation Oncology, Cancer Hospital and Institute, Peking Union Medical College (PUMC) and Chinese Academy of Medical Sciences (CAMS)

Clinical research coordinator: Jun Liang and Nan Bi

Study Outline 3
1. Background 5
2. Purpose 6
3. Eligibility 6
3.1 Inclusion Criteria 6
3.2 Exclusion Criteria 6
3.3 Criteria for Discontinuation of Protocol Treatment 7
4. Study Design 7
4.1 Study Endpoint 8
4.2 Evaluations Before, During and After Treatment 8
4.3 SNP Genotyping 9
4.4 Radiation Therapy 9
4.5 Chemotherapy 10
5. Patients Assessment and Follow Up 12
5.1 Response Assessment 12
5.2 Adverse Event Evaluation 13
5.3 Follow Up after Treatment 13
6. Statistical Considerations 13
6.1 Hypothesis 13
6.2 Sample Size and Power 14
6.3 Statistical Designs 14
7. Reference 14

Study Outline

Study title: Cisplatin/Etoposide and Concurrent Radiotherapy With or Without Celecoxib in Patients With Unresectable Locally Advanced Non-small Cell Lung Cancer (NSCLC)

Type of study: The physician initiated phase II randomized clinical trial

Participants: Patients with unresectable stage III NSCLC confirmed with cytology or histology were eligible.

Primary endpoint: Overall Survival (OS)

Secondary endpoints: Treatment-related toxicities, progression-free survival (PFS), and to evaluate the survival benefit of CCRT+C arm compared to CCRT arm in patients with COX-2 high-risk genotype.

Study design: This is a single-institution, open-label, randomized phase II trial

Expected number of participants: 50 patients in the test group, 50 patients in the control group

Inclusion criteria:

- 18-70 years old, male or female
- Histological or cytological confirmed stage III NSCLC
- Eastern Cooperative Oncology Group (ECOG) performance status ≤1
- ≤10% weight loss in the 3 months before inclusion
- inoperable AJCC stage IIIA, or IIIB
- Normal organ function

Exclusion Criteria:

- active uncontrolled infection
- clinically significant cardiovascular disease
- history of other malignancies
- forced expiratory volume in 1 s <40% of normal
- previous treatment with radiotherapy, chemotherapy or immunotherapy

Duration of study:

Start time: 12/2011

Expected end time: 11/2015

Radiation Therapy:

Target Volume: The definitions of volumes will be in accordance with the standard protocol of simplified intensity-modulated radiotherapy (sIMRT).

Dose: A dose of 60 Gy (2 Gy per fraction) started on the first day of chemotherapy.

Treatment technique: sIMRT.

Chemotherapy

1. Cisplatin/Etoposide: 50 mg/m2/d of cisplatin on days 1, 8, 29, and 36 and 50 mg/m2/d of etoposide on days 1–5 and 29–33. All the concurrent chemotherapy agents are administrated intravenously.
2. Celecoxib: 200mg twice daily was started one week before initiation of radiotherapy and was continued without interruption until the end of radiation therapy.

Follow-up duration: 5 years

1. Background

Approximately 30% of patients with non–small cell lung cancer (NSCLC) have locally advanced diseases (LA-NSCLC) [1]. Although the concurrent chemotherapy and radiation (CRT) are considered the standard care [2], novel agents are needed to improve therapeutic efficacy and selectively reduce normal tissue injury.

Overexpression of cyclooxygenase-2 (COX-2) has been reported in NSCLC [3] [4]. Increased COX-2 expression is associated with more aggressive tumor behavior and poor prognosis in NSCLC patients [5]. COX-2 may also play a part in patient survival after ionizing radiation [6-8]. The preclinical findings suggested that COX-2 inhibitors might potentially improve radiotherapy or chemoradiotherapy. A phase I clinical trial demonstrated that the selective COX-2 inhibitor celecoxib can be safely administered concurrently with thoracic radiotherapy [9]. Additionally, selective COX-2 inhibitors are used as a type of nonsteroidal anti-inflammatory drug (NSAID) and it is generally believed that inflammation significantly participates in the pathogenesis of radiation injury [10]. Therefore, COX-2 inhibitor celecoxib might provide a reduction in radiation-induced lung toxicity, which is dose-limiting toxicity for lung cancer.

It is critical to identify patients who may benefit from COX-2 target therapy. We previously reported that −1195G/A SNP (rs689466) in the COX-2 promoter region, is associated with a different survival advantage in inoperable locally advanced NSCLC treated with chemoradiation or radiation alone [8]. Tumors carrying unfavorable −1195AA genotype were more radiation resistant than those with the −1195GA + GG genotypes and need intensive treatment. Functional study showed that the −1195G to A change creates a c-MYB binding site and, thus, displays a higher promoter activity. Compared with the −1195G-containing counterparts, the

−1195AA carriers showed significantly increased COX-2 expression in vitro and in vivo.

On the basis of these results, here we design a randomized phase II clinical trial, which tries to evaluate the value of combined selective COX-2 inhibition with standard concurrent chemoradiation therapy (CCRT) for patients with unresectable stage III NSCLC.

2. Purpose

To determine the value of combined selective COX-2 inhibition with standard concurrent chemoradiation therapy (CCRT) for patients with unresectable stage III NSCLC, with a focus on survival, treatment-related lung toxicity, and the prediction role for the COX-2 −1195G/A polymorphism.

3. Eligibility

3.1 Inclusion Criteria

- 18-70 years old, male or female
- Histological or cytological confirmed stage III NSCLC
- Eastern Cooperative Oncology Group (ECOG) performance status ≤1
- ≤10% weight loss in the 3 months before inclusion
- inoperable AJCC stage IIIA, or IIIB
- Normal organ function

3.2 Exclusion Criteria

- active uncontrolled infection

- clinically significant cardiovascular disease
- history of other malignancies
- forced expiratory volume in 1 s <40% of normal
- previous treatment with radiotherapy, chemotherapy or immunotherapy

3.3 Criteria for Discontinuation of Protocol Treatment

- Patient’s refusal.
- A delay in protocol treatment of greater than 2 weeks.
- Protocol treatment is discontinued for non-tumor reasons
- In the opinion of the Investigator, indicates that continued treatment with all study therapy is not in the best interest of the participant.

4. Study Design

This is a single-institution, open-label, randomized phase II trial of celecoxib administered concurrently with cisplatin, etoposide, and radiation therapy in patients with locally advanced NSCLC, to determine the feasibility, activity, and toxicity of this combination on unresectable NSCLC, and further to examine biomarkers to predict response to the treatment.

	Treatment protocols
The control group (CCRT arm)	60Gy of thoracic radiation therapy concurrent with etoposide 50mg/m2 on days 1–5 and cisplatin 50mg/m2 on days 1 and 8 every 4 weeks for two cycles alone
Test group (CCRT+C arm)	The exact same treatment with control group + celecoxib (200mg twice daily was started one week before initiation of radiotherapy

	and was continued without interruption until the end of radiation therapy)

4.1 Study Endpoint

- Primary endpoint: overall survival
- Secondary endpoints: treatment-related toxicities, progression-free survival (PFS), and to evaluate the survival benefit of CCRT+C arm compared to CCRT arm in patients with COX-2 high-risk −1195AA genotype

4.2 Evaluations Before, During and After Treatment.

4.2.1 Pretreatment evaluation (within 1 week prior to treatment)

- H&P
- Performance scoring
- Blood count, liver and renal function tests, and electrolyte test
- Pulmonary function test
- ECG
- Bronchoscopy
- Chest and abdominal CT
- Brain MRI or CT
- Radionuclide bone scan
- Adjunctive use of chest MRI or FDG positron emission tomography (FDG-PET) when available

4.2.2 Evaluation during treatment

- H&P
- Toxicity evaluation based on CTC 3.0 criteria every week

- Necessary test to evaluate disease or toxicities
- QOL analysis at the end of the 4th week

4.2.3 Evaluation after treatment

- H&P
- Chest CT and abdominal CT/ultrasound
- QOL analysis at the end of treatment
- Response evaluation at four weeks after treatment

4.3 SNP Genotyping

Genomic DNA was extracted from a 5-mL blood sample that was collected in a blinded manner at baseline. Each specimen was stored at -80°C. The COX-2−1195G/A polymorphisms were genotyped using Sequenome MassArray method as previously described [8]. The high-risk genotype was −1195AA homozygote, and low-risk group included −1195GA and −1195GG genotypes.

4.4 Radiation Therapy

4.3.1 Target Volumes

1) Definition of the GTV: the gross tumor volume (GTV) includes the primary disease as well as any involved regional lymph nodes, which are defined as those with a short-axis diameter of at least 1 cm on CT scan or with a short-axis diameter of less than 1 cm but with high fluorodeoxy-glucose (FDG) uptake on PET-CT scan. The primary tumor is contoured using pulmonary window CT settings and nodal GTV using the mediastinal window. The use of PET or MRI to distinguish tumor from fluid/atelectasis is encouraged.

2) Definition of the CTV: the clinical tumor volume (CTV) is defined to be the primary tumor plus a 0.6 cm to 0.8 cm margin, ipsilateral hilum, subcarinal, and the ipsilateral mediastinal to the highest lymph node stations involved. Elective treatment of the mediastinum and supraclavicular fossae will not be done.
3) Definition of the PTV: the PTV includes the CTV plus a total margin of at least 0.5 cm.
4) All of the patients underwent simplified intensity-modulated radiotherapy (sIMRT).

4.3.2 Radiation Dose

1) The total dose will be 60Gy in 30 fractions. Patients will receive treatment 5 days per week, in once daily fractions, 2 Gy per fraction.
2) Normalization of the treatment plan will cover 95% of the PTV with the prescription dose. The target dose uniformity should be within +7% and -7%. Inhomogeneity corrections will be used when radiation doses are calculated.
3) The maximum spinal cord dose should not exceed 45 Gy at any point. The mean lung dose (MLD) should be less than 17Gy. The lung volume receiving >20 Gy (V20), which is calculated by using total lung volume minus GTV, is limited to no more than 30%. The lung volume receiving >30 Gy (V30) is no more than 20%.

4.5 Chemotherapy

1) Cisplatin/Etoposide: 50 mg/m2/d of cisplatin on days 1, 8, 29, and 36 and 50 mg/m2/d of etoposide on days 1–5 and 29–33

2) Celecoxib: 200mg twice daily was started one week before initiation of radiotherapy and was continued without interruption until the end of radiation therapy

3) All the concurrent chemotherapy agents are administrated intravenously.

4) Etoposide/cisplatin and Celecoxib dose modifications for Treatment-related toxicity

	**Etoposide**	**Cisplatin**
**Hematologic toxicity**		
ANC>1000/mm^3	100％	100％
ANC 500-999/mm^3	75％	75％
ANC <500/mm^3	Hold treatment until recovery	Hold treatment until recovery
PLT>80000/mm^3	100％	100％
PLT 50000-79000/mm^3	75％	75％
PLT<50000/mm^3	Hold treatment until recovery	Hold treatment until recovery
ANC1000/mm^3 and fever≥38°	Hold treatment until recovery	Hold treatment until recovery
**Neuralgia/muscular pain/ arthralgia**		
Grade 0-1	100％	100％

Grade 2	75％	75％
Grade 3 or more	Hold treatment until recovery	Hold treatment until recovery
**<u>Esophagitis</u>**		
Grade 0-2	100％	100％
Grade 3	75％	75％
Grade 4 or more	Hold treatment until recovery	Hold treatment until recovery

5. Patients Assessment and Follow Up

5.1 Response Assessment

The response will be evaluated using RECIST criteria.

Response Criteria: Evaluation of target lesions

Complete Response (CR)	Disappearance of all target lesions
Partial Response (PR)	At least a 30% decrease in the sum of the LD of target lesions, taking as reference the baseline sum LD
Stable Disease (SD)	Neither sufficient shrinkage to qualify for PR nor sufficient increase to qualify for PD, taking as reference the smallest sum LD since the treatment

	started
Progressive Disease (PD)	At least a 20% increase in the sum of the LD of target lesions, taking as reference the smallest sum LD recorded since the treatment started or the appearance of one or more new lesions

LD: Longest Diameter

5.2 Adverse Event Evaluation

Treatment-related toxicities would be evaluated using NCI CTCAE, v4.0.

5.3 Follow Up after Treatment

The patients will be followed up every 3 months from hospital medical records and/or by phone. The follow-up evaluations should consist of a history, physical examination, and a thoracic CT at intervals of 3 months or earlier if clinically indicated. Other imaging examinations will be obtained when recurrence is suspected.

6. Statistical Considerations

6.1 Hypothesis

Adding celecoxib, a COX-2 inhibitor to the concurrent chemoradiation may improve the OS without increasing or even reducing lung toxicity compared to concurrent chemoradiation only. And patients with the high-risk genotype would benefit the most from COX-2 blockade.

6.2 Sample Size and Power

The primary endpoint was overall survival (OS). We reported a 2-year OS rate of 48% for the EP based CCRT [11]. Liao et al. reported a 2-year OS rate of 67% with combined CCRT with celecoxib [9]. The power analysis and sample size estimation were completed using the log-rank test. Assuming 10% of patients would be lost at follow-up, with the proposed sample size of 50 subjects per arm, it provides 70% power to detect 19% superiority (67% vs. 48%) in OS at two years from randomization with a two-sided type I error rate of 0.05. The secondary endpoints were treatment-related toxicities, progression-free survival (PFS), and to evaluate the survival benefit of CCRT+C arm compared to CCRT arm in patients with COX-2 high-risk −1195AA genotype.

6.3 Statistical Designs

Survival rates were calculated from the day of randomization and estimated using the Kaplan-Meier method. The difference between two arms was estimated using student’s t-test, nonparametric Mann ‒ Whitney u test or χ^2-test. Hazard ratios (unadjusted and adjusted) were estimated using Cox proportional hazards models. Toxicity rates and response rates were compared by Fisher’s exact test. A two-sided $P<0.05$ was considered statistically significant.

7. Reference

1. Yang P, Allen MS, Aubry MC, et al: Clinical features of 5,628 primary lung cancer patients: experience at Mayo Clinic from 1997 to 2003. Chest 128:452-62, 2005.

2. Auperin A, Le Pechoux C, Rolland E, et al: Meta-analysis of concomitant versus sequential radiochemotherapy in locally advanced non-small-cell lung cancer. J Clin Oncol 28:2181-90, 2010

3. Hida T, Yatabe Y, Achiwa H, et al. Increased expression of cyclooxygenase 2 occurs frequently in human lung cancers, specifically in adenocarcinomas. Cancer Res 1998;58:3761–4.

4. Wolff H, Saukkonen K, Anttila S, et al. Expression of cyclooxygenase-2 in human lung carcinoma. Cancer Res 1998;58:4997–5001.

5. Achiwa H, Yatabe Y, Hida T, et al. Prognostic significance of elevated cyclooxygenase 2 expression in primary, resected lung adenocarcinomas. Clin Cancer Res 1999;5:1001–5.

6. O'Connor JK, Avent J, Lee RJ, et al. Cyclooxygenase-2 expression correlates with diminished survival in invasive breast cancer treated with mastectomy and radiotherapy. Int J Radiat Oncol Biol Phys 2004;58:1034–40.

7. Gaffney DK, Holden J, Davis M, et al. Elevated cyclooxygenase-2 expression correlates with diminished survival in carcinoma of the cervix treated with radiotherapy. Int J Radiat Oncol Biol Phys 2001;49:1213–7

8. Bi N, Yang M, Zhang L, et al. Cyclooxygenase-2 genetic variants are associated with survival in unresectable locally advanced non-small cell lung cancer. Clin Cancer Res. 2010;16(8):2383-90.

9. Liao Z, Komaki R, Milas L, Yuan C, Kies M, Chang JY, et al. A phase I clinical trial of thoracic radiotherapy and concurrent celecoxib for patients with unfavorable performance status inoperable/unresectable non-small cell lung cancer. Clin Cancer Res. 2005 May 1;11(9):3342-8.

10. Hunter NR, Valdecanas D, Liao Z, Milas L, Thames HD, Mason KA. Mitigation and treatment of radiation-induced thoracic injury with a cyclooxygenase-2 inhibitor, celecoxib. Int J Radiat Oncol Biol Phys. 2013 Feb 1;85(2):472-6.

11. Liang J, Bi N, Wu S, et al. Etoposide and Cisplatin vs Paclitaxel and Carboplatin With Concurrent Thoracic Radiotherapy in Unresectable Stage III Non-Small Cell Lung Cancer: A Multicenter Randomized Phase III Trial. Ann Oncol. 2017;28(4):777-783.
