# Supplementary material for: Effect of Concurrent Chemoradiation With Celecoxib vs Concurrent Chemoradiation Alone on Survival Among Patients With Non–Small Cell Lung Cancer With and Without Cyclooxygenase 2 Genetic Variants: A Phase 2 Randomized Clinical Trial
Source: JAMA Netw Open. 2019 Dec 18;2(12):e1918070. doi: 10.1001/jamanetworkopen.2019.18070 (PMC6991217; doi:10.1001/jamanetworkopen.2019.18070)
Supplement: Supplement 2. — eTable. Patient Characteristics for COX-2 Genotyping eFigure 1. Kaplan-Meier Curves by Treatment Arm eFigure 2. Kaplan-Meier Curves by Gene Testing eFigure 3. Kaplan-Meier Curves in Patients Without EGFR Mutations [file jamanetwopen-2-e1918070-s002.pdf]

Supplementary Online Content

Bi N, Liang J, Zhou Z, et al. Effect of concurrent chemoradiation with celecoxib vs concurrent chemoradiation alone on survival among patients with non–small cell lung cancer with and without cyclooxygenase 2 genetic variants: a phase 2 randomized clinical trial. *JAMA Netw Open*. 2019;2(12):e1918070. doi:10.1001/jamanetworkopen.2019.18070

eTable. Patient Characteristics for COX-2 Genotyping
eFigure 1. Kaplan-Meier Curves by Treatment Arm
eFigure 2. Kaplan-Meier Curves by Gene Testing
eFigure 3. Kaplan-Meier Curves in Patients Without *EGFR* Mutations

This supplementary material has been provided by the authors to give readers additional information about their work.

© 2019 Bi N et al. *JAMA Network Open.*

eTable 1. Patient Characteristics for *COX-2* Genotyping

Patient characteristic	With genotyping (*n* = 60)	Without genotyping (*n* = 36)	*P* value
Age			1.00
< 65, y	53 (80.3%)	24 (80.0%)	
≥ 65, y	13 (19.7%)	6(20.0%)	
Gender			0.05
Male	54(81.8%)	19 (63.3%)	
Female	12 (18.2%)	11 (36.7%)	
ECOG			0·20
0	38(57.6%)	13 (43.3%)	
1	28 (42.4%)	17 56.7%)	
Pathology			0·63
Squamous	48 (72.7%)	19 (63.3%)	
Adenocarcinoma	15(22.7%)	9 (30%)	
Other	3 (4.5%)	2(6.7%)	
AJCC stage			0.57
IIIA	26(39.4%)	10(33.3%)	
IIIB	40 (60.6%)	20(66.7%)	
Smoking history			0.15
Yes	53 (80.3%)	20 (66.7%)	
No	13 (19.7%)	10 (33.3%)	

Abbreviations: CCRT, concurrent chemoradiation; CCRT+C, concurrent chemoradiation and celecoxib; ECOG, Eastern Cooperative Oncology Group; AJCC, American Joint Committee on Cancer.

© 2019 Bi N et al. *JAMA Network Open.*

eFigure 1. Kaplan-Meier Curves by Treatment Arm

(A) OS and (B) PFS.

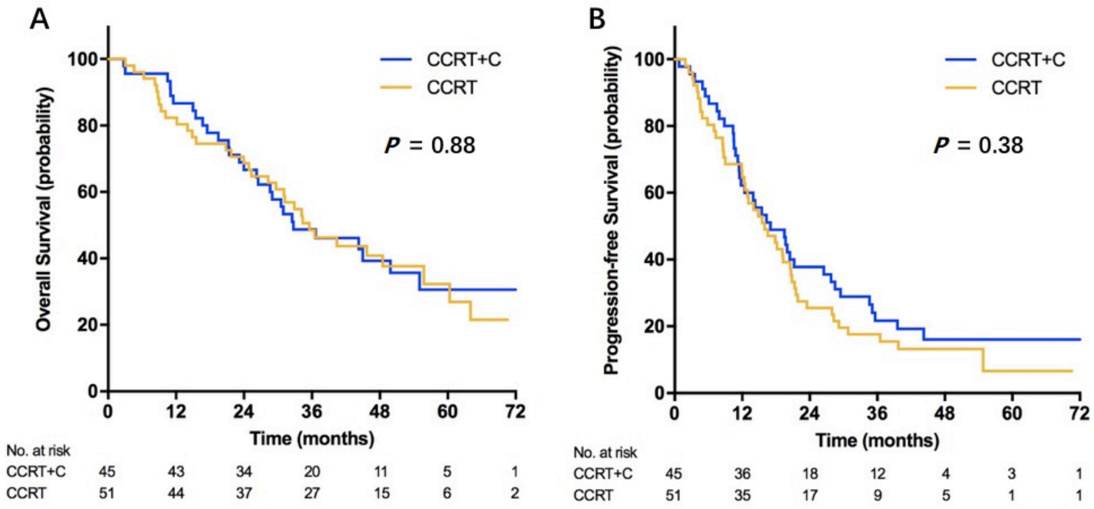

© 2019 Bi N et al. *JAMA Network Open.*

eFigure 2. Kaplan-Meier Curves by Gene Testing

(A) OS and (B) PFS.

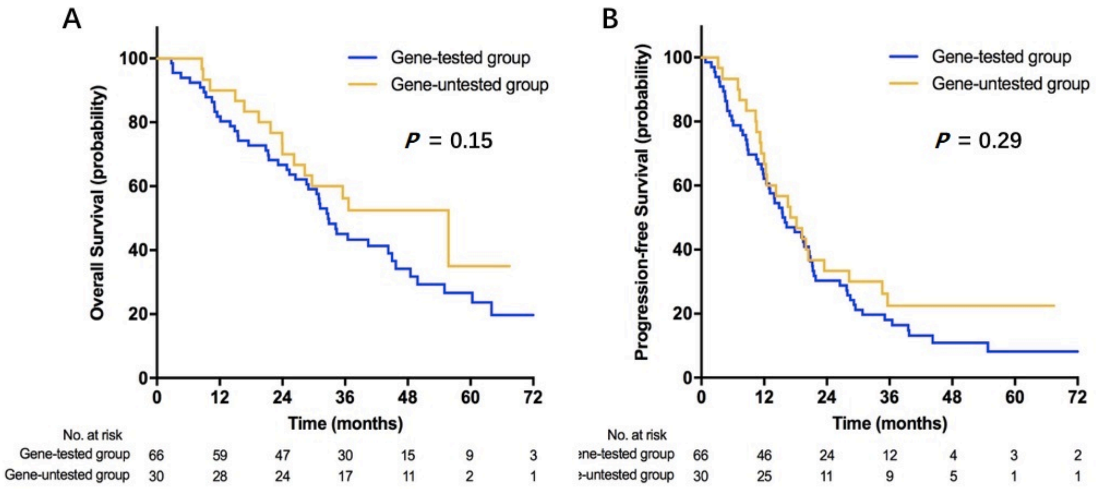

© 2019 Bi N et al. *JAMA Network Open.*

eFigure 3. Kaplan-Meier Curves in Patients Without *EGFR* Mutations

(A) OS and (B) PFS by celecoxib in patients with high-risk genotype. (C) OS and (D) PFS by celecoxib in patients with low-risk genotype.

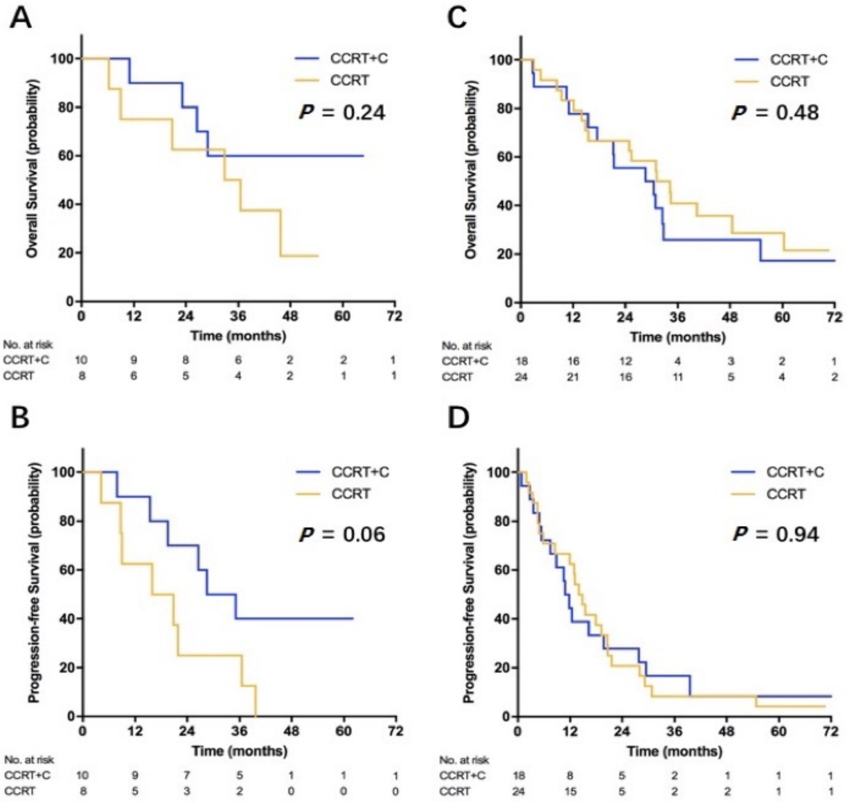

© 2019 Bi N et al. *JAMA Network Open.*
