# Supplementary material for: Effect of Concurrent Chemoradiation With Celecoxib vs Concurrent Chemoradiation Alone on Survival Among Patients With Non–Small Cell Lung Cancer With and Without Cyclooxygenase 2 Genetic Variants: A Phase 2 Randomized Clinical Trial
Source: JAMA Netw Open. 2019 Dec 18;2(12):e1918070. doi: 10.1001/jamanetworkopen.2019.18070 (PMC6991217; doi:10.1001/jamanetworkopen.2019.18070)
Supplement: Supplement 3. — Data Sharing Statement [file jamanetwopen-2-e1918070-s003.pdf]

Data Sharing Statement

Bi. Effect of Concurrent Chemoradiation With Celecoxib vs Concurrent Chemoradiation Alone on Survival Among Patients With Non-Small Cell Lung Cancer With and Without Cyclooxygenase 2 Genetic Variants. *JAMA Netw Open*. Published December 18, 2019.
10.1001/jamanetworkopen.2019.18070

Data
Data available: No
